# Tannin: An Insight into its Cosmeceutical Properties and Uses

**DOI:** 10.1002/gch2.202500115

**Published:** 2025-04-27

**Authors:** Rosaria Ciriminna, Giovanna Li Petri, Giuseppe Angellotti, Enrica Fontananova, Francesco Meneguzzo, Rafael Luque, Mario Pagliaro

**Affiliations:** ^1^ Istituto per lo Studio dei Materiali Nanostrutturati CNR via U. La Malfa 153 Palermo 90146 Italy; ^2^ Istituto per la Tecnologia delle Membrane CNR via P. Bucci, cubo 17/c Rende (CS) 87036 Italy; ^3^ Istituto per la Bioeconomia CNR via Madonna del Piano 10 Sesto Fiorentino FI 50019 Italy; ^4^ Universidad Espíritu Santo (UEES) Samborondón EC091650 Ecuador

**Keywords:** hair relaxer, natural cosmetics, taninoplastia, tannic acid, tannin

## Abstract

Little scholarly research has been published on the cosmetic uses of tannins. This study identifies the emerging uses of tannin as cosmeceutical ingredient in skin and hair care products. A green chemistry, health, and economic perspective is offered on future developments concerning the use of tannins in cosmetic products massively employed such as hair dyes and hair relaxers. Besides filling a gap in the literature, the study will hopefully accelerate the uptake of tannins as cosmeceutical ingredients in cosmetic products widely used worldwide such as hair dyes and hair relaxers, eventually replacing harmful chemicals with a class of natural products imparted with broad‐scope health‐beneficial properties.

## Graphical abstract

Filling a gap in the literature, this study identifies the emerging uses of tannin as cosmeceutical ingredient in skin and hair care products. The account offers also a green chemistry, health and economic perspective on future developments concerning the use of tannins in cosmetic products widely employed worldwide such as hair dyes and hair relaxers.

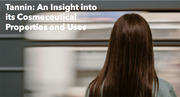

## 1 Introduction

Tannin is an old natural product consisting of a mixture of oligomeric polyphenolic compounds (molecular mass 500–3000 g mol^−1^) commercially sourced from wood and wood bark.^[^ ^1^ ^]^ Including gallotannins and ellagitannins (polyesters of gallic and hexahydroxydiphenic acid, respectively), hydrolyzable (or hydrolyzing) tannins are generally sourced from sweet chestnut and oak wood and bark. Comprising over 90% of the world's tannin output, condensed (non‐hydrolyzable) polyflavonoid tannins (chatechins; namely condensed flavans containing no sugar residues) are sourced from mimosa, quebracho, and acacia woods and barks. Condensed tannins are also abundant in fruits such as grapes, and apples, and leaves such as tea leaves.^[^ ^1^ ^]^

Traditionally employed in the leather industry due to its binding with collagen through hydrophobic and H‐bonds interactions,^[^ ^2^ ^]^ tannin stabilizes tanned hide (leather) against microbial and oxidative degradation thanks to the inhibition of proteolytic enzymes.

Other traditional commercial uses of tannins are in winemaking (tannin is added as a clarifying agent and stabilizer), and as raw material replacing phenol in the production of phenol‐formaldehyde resins.^[^ ^1^ ^]^ New applications of chestnut tannin in animal feed and in agriculture emerged in the first two decades of the 2000s,^[^ ^3^ ^]^ while pharmacological uses of both hydrolyzable and condensed tannins are now in sight.^[^ ^4^ ^]^

Concluding in 2021 a bioeconomy perspective on the emerging new uses of tannin we wrote that “flourishing research on tannin's biological activity and technological applications has revealed many new properties that are likely to drive significant growth in demand in the near and mid‐term future.”^[^ ^3^ ^]^ Driven by the aforementioned new applications, in the subsequent five years said near‐term significant growth actually took place. The global production of both hydrolyzable and condensed tannins historically standing a 230 000 tonnes per year,^[^ ^3^ ^]^ significantly expanded.

Consumed by more than 80% of the world population in one or another form either through beverages (tea, coffee, wine, beer, etc.), chocolate, seeds, leaves, fruits, cereals, legumes, herbs, and spices, tannin in general is a health beneficial substance. Daily intake of tannin below the range of 1.5–2.5 g is safe for consumption and does not cause any side effects.^[^ ^5^ ^]^ Tannin's powerful antioxidant, anticancer, antiallergic and anti‐inflammatory, astringent, and antimicrobial properties made tannin extracts widely employed in traditional medicine.^[^ ^6^ ^]^ Though soluble in water and exerting powerful antioxidant and ant‐inflammatory activity, tannin historically has not been used in cosmetics.

The rediscovery of natural products in cosmetics in the course of the 1980s along with the concomitant advent of cosmeceuticals originated the first niche demand of tannins also in cosmetics. Introduced by Kilgman in 1984, the term “cosmeceutical” indicates a substance “in between a drug and a cosmetic. A cosmeceutical does something more than coloring the skin and something less than a therapeutic drug.”^[^ ^7^ ^]^ Twenty years later, Kilgman insisted that academic research centers specializing in dermatology should “realize that cosmeceuticals are important and worthy of quality research.”^[^ ^7^ ^]^

Little (though important) scholarly research has been published on the cosmetic uses of tannins. In 2013, Bylka and coworkers in Poland wrote in a cosmetology journal that tannins were used topically in the treatment of skin inflammation and abrasions as well as an ingredient of shampoos for dark and greasy hair, sometimes in anti‐aging.^[^ ^8^ ^]^ In 2020, academic and industrial research chemists based in Germany,^[^ ^9^ ^]^ and academic researchers in China^[^ ^10^ ^]^ reported that tannin in combination with metals can be successfully used as hair dye.

Previously, in 2012, the first commercial application of a tannins mixture as a hair relaxer was patented in Brazil with the new trademark of “Taninoplastia” (see below).^[^ ^11^ ^]^ Finally, in early 2024 Li and coworkers in China reviewing the biobased hair dyes included the aforementioned works describing the use of tannic acid in combination with metal ions.^[^ ^12^ ^]^

Filling a gap in the literature, this study identifies the emerging uses of tannin as cosmeceutical in skin and hair care. We conclude offering a green chemistry, health and economic perspective on future developments concerning the use of tannins in cosmetic products massively used such as hair dyes and hair relaxers.

## 2 Tannin in Skin Care

Often presented as penta‐*m*‐digalloyl glucose, consisting of a glucose molecule esterified at all five hydroxyl moieties with two galloyl (3,4,5‐ trihydroxyphenyl) units, tannic acid is present in many personal care and skin care products. For example, TA is present in one bar soap product, three facial moisturizer/treatment products, one mask product, one around‐eye cream product, five serums and essences products, two moisturizer products, five facial cleanser products, one daily use formulation with sun protection factor, and one shampoo product commercialized in the USA.^[^ ^13^ ^]^

**Figure 1:**
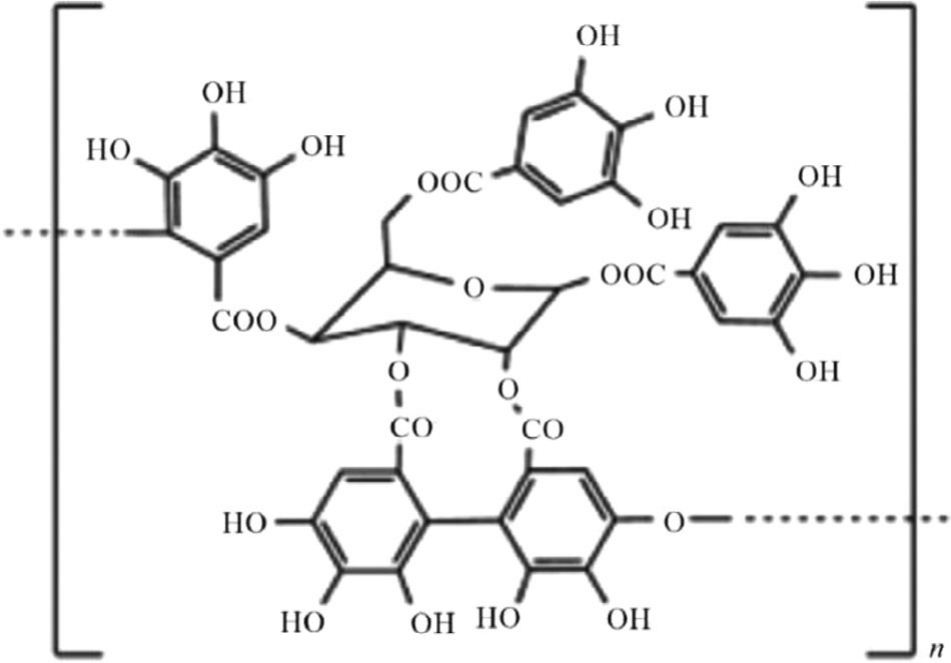
Structure of pentagalloylglucose repeating unit undergoing oligomerization in *n* monomers.

Actually, so‐called tannic acid generally obtained from sweet chestnut (*Castanea sativa* Mill.) aqueous extract, is a typical product containing hydrolyzable tannins consisting of a mixture of ellagitannins whose varying composition is actually due to the hydrolysis of the real structure of hydrolyzable tannin in plants: a high molecular weight random series of pentagalloylglucose oligomers of the repeating unit (**Figure** **1**).^[^ ^14^ ^]^

Cosmetics and personal care products are not required to be tested for safety before being allowed on the market. As of early 2025, Environmental Working Group, an environmental association based in the USA, assigned to TA a “Skin Deep ingredient hazard score” of 1 (“fair”).^[^ ^15^ ^]^ The EWG “Skin Deep” scoring system (an index from 1 to 10) reflects known and suspected hazards linked to the ingredients, and aims to help the public understand whether a product is safe to use or whether it contains ingredients of concern. Added in 2% amount to a formulation aptly called “Tannic serum,”^[^ ^16^ ^]^ TA helps to reduce the look of age spots and relieves redness.

Tannic acid protects the skin from inflammation caused by external irritation, as shown in 2017 by Nakamura and coworkers in Japan who evaluated the effects of TA using a mouse 12‐O‐tetradecanoylphorbol 13‐acetate (TPA)‐induced skin inflammation model and a reconstructed human epidermal model.^[^ ^17^ ^]^ TA treatment of the skin prevented Lucifer Yellow dye from permeating the skin, with TA acting as a barrier against external stimulants such as TPA and artificial sweat on the stratum corneum (SC) surface.

In 2020, Lautenschlager and coworkers in Brazil reported potent antioxidant, anti‐collagenase, and anti‐elastase activities, UV‐absorption effects, and the ability in prevent oxidative stress, oxidative damages, and MMP‐1 induction in UVB‐irradiated L929 fibroblasts protected by TA.^[^ ^18^ ^]^ In detail, the team showed that at lower doses TA exhibited effective protection against UVB‐induced cell viability decrease. Furthermore, TA was able to prevent partially the morphological alterations induced by UVB exposure, confirming its cytoprotection results. Moreover, TA treatment prevented cellular photodamage and subsequently photoaging, by inhibiting lipid peroxidation, depolarization of mitochondrial transmembrane potential, DNA damage, and MMP‐1 expression, a protein closely related to the structural degeneration of the dermis extracellular matrix. Although GA and TA exhibited similar inhibitory activity toward collagenase, TA was more potent in inhibiting elastase. In addition, TA presented a broader UV absorption spectrum. Furthermore, TA treatment in UVB‐irradiated cells attenuated redox imbalance, as observed by its ability to inhibit ROS production, NADPH oxidase activation, and depletion of the endogenous antioxidant defense system. In general, TA was more effective than GA in scavenging radicals DPPH•, superoxide anion, peroxyl, nitric oxide, and peroxynitrite, and to reduce ferric ions.

**Figure 2:**
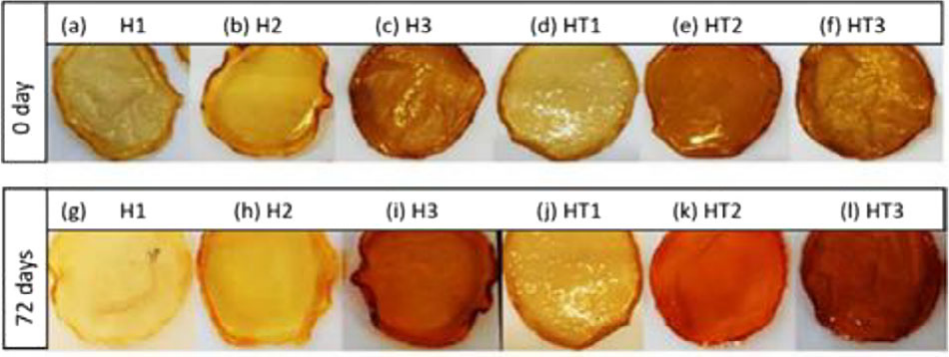
Hydrogel films with ca. 2 mm thickness without a–c, g–i) and with (d–f, j–l) TA observed on 0 (a–f) and 72 (g–l) days after preparation. [Reproduced from ref. [19], Creative Commons License CC BY 4.0].

In 2024, Gonçalves and coworkers also based in Brazil reported that hydrogel films (**Figure** **2**) based on gelatin, glutaraldehyde and glycerol functionalized with tannic acid as anti‐aging active, had excellent mechanical and antiaging properties suitable for skin care.^[^ ^19^ ^]^

Among the investigated properties, the H2 and HT2 hydrogel films showed the best results and were selected for the tests. The release of the compound from the films occurred slowly as shown via the Folin–Ciocalteu method and the ferric chloride test. The 1 wt.% addition of TA caused a reduction in the tensile strength, Young's modulus, and puncture strength. Despite this decrease, the values remainede acceptable for dermatological use.

In general, as noted by Jing and coworkers in 2022, numerous studies suggest that TA is useful for the external treatment of skin inflammation.^[^ ^20^ ^]^ Tannic acid can regulate multiple signaling pathways through several targets. For example, in human HaCaT keratinocytes UVB‐induced interleukin‐18 production is downregulated by tannic acid.^[^ ^21^ ^]^

## 3 Tannin in Hair Care

The use of both condensed and hydrolyzable tannins for hair‐protecting cosmetic formulation goes back to the early 1980s when a Japanese cosmetic company patented their use “to suppress the separation of amino acids caused by the damage of hair.”^[^ ^22^ ^]^ The patented cosmetic composition preferably contained 1–5 wt.% of a condensed tannin having a molecular weight of 500‐3,500 g mol^−1^. Though patented by a large cosmetic and personal care company based in Japan, the commercial use of tannins for hair protection as well as straightening agents had to wait the early 2010s, when a cosmetic company based in Brazil filed a patent application protecting the intellectual property of the process in 2012.^[^ ^23^ ^]^

The process was subsequently tradenamed “Taninoplastia”.^[^ ^24^ ^]^ The aforementioned cosmetic company based in Brazil launched its first product for carrying out the “Taninoplastia” shortly afterward.

Conventional straighteners used to straighten kinky, curly, or even wavy hair such as hydroxides, thiols, or hydrolyzed keratin solution (harmful glyoxylic acid or formaldehyde are not primarily straighteners, they are used as the heat of the dryer volatilizes the formaldehyde increasing the fixation capacity of the hydrolyzed keratin solution in the natural keratin of the hair), typically work breaking the disulfide (S‐S) bond in keratin. As a result, numerous S‐H thiol groups are formed with the result that the hair is mechanically weakened becoming “manageable” (straight shaped by applying brushing).^[^ ^25^ ^]^

To the best of our knowledge, no research reports describe the interaction between condensed tannin molecules in these formulations and keratin. However, thiol groups (‐SH) contained in keratine's cysteine residues are well known to react with small tannin molecules such as epigallocatechin gallate (EGCG, a tannin being the major catechin found in green tea) to form covalent bonds. For example, reacting human hair keratin with EGCG, Li, and coworkers in China reported in 2021 the appearance in the XPS profile of the product of the sulfur oxidized signal only indicating that all thiol groups in keratin react with EGCG.^[^ ^26^ ^]^

In brief, the mechanism is symmetrical with respect to the typical hair straightening cream comprised of dialdehyde, surfactant, emollient, emulsifier, preservative, diluent, and skin‐protecting agent. Thiol groups are replaced by S‐tannin moieties that both reinforce and make the air manageable. Indeed, further proof that another tannin (tannic acid) binds to keratin, was reported by Lee and coworkers in 2025.^[^ ^27^ ^]^

**Figure 3:**
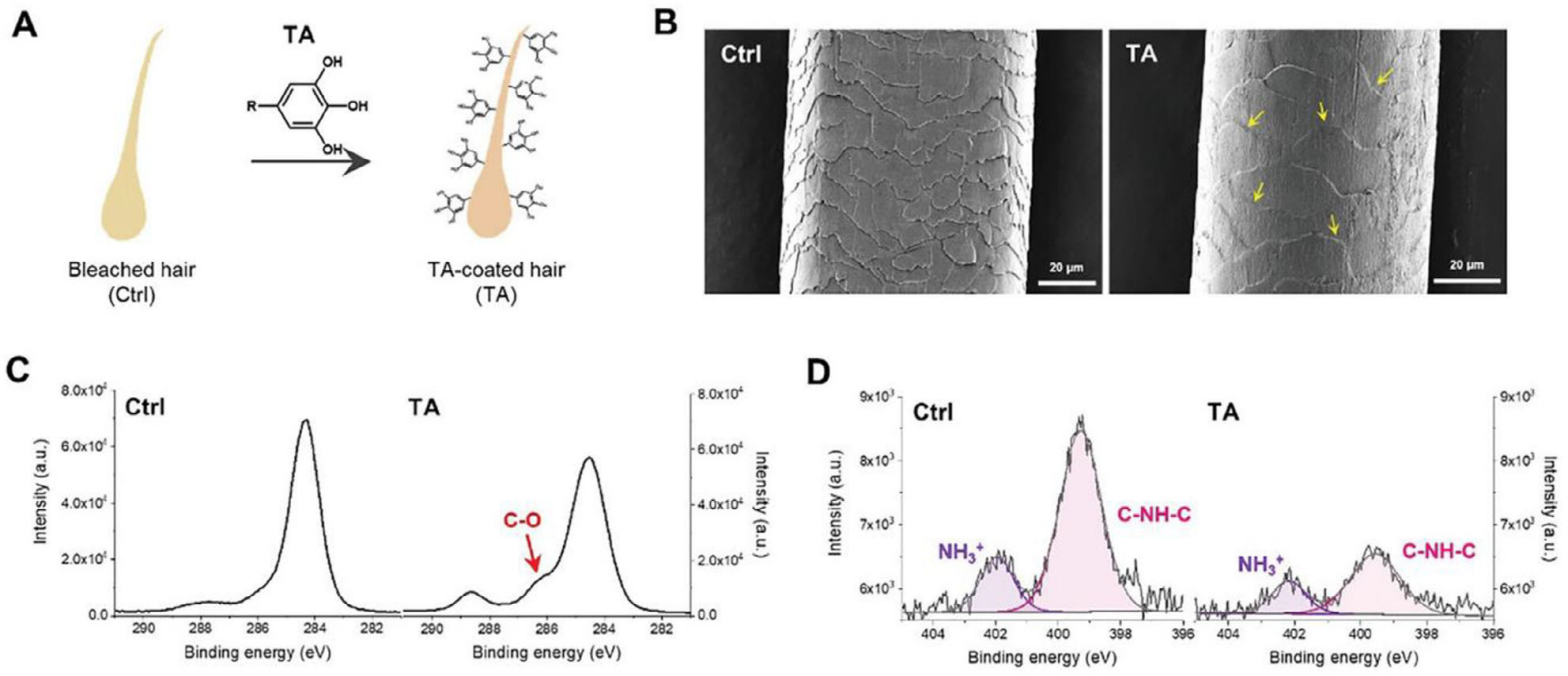
A) Schematic illustration of tannic acid coating on human hair strands. B) SEM images of untreated human hair (left) and tannic acid‐coated hair (right), with yellow arrows indicating the blurred layer‐to‐layer cuticle boundary. (Scale bar: 20 µm) C) C1s XPS spectra of untreated (left) and tannic acid‐coated hair (right), with the red arrow highlighting the presence of C─O bonds. D) N1s XPS spectra of untreated (left) and tannic acid‐coated hair (right). [Reproduced from ref. [27], Creative Commons License CC BY 4.0].

Bleached hair was immersed in a tannic acid solution (16 mg mL^−1^), rinsed with water, then dried (**Figure** **3**A). Scanning electron microscopy (SEM) revealed that the tannic acid‐coated hair exhibited a blurred layer‐to‐layer cuticle boundary, in contrast to the distinct boundary seen in uncoated hair. Confirming successful coating on the hair surface, the XPS analysis of the C1s spectrum for the TA‐coated hair showed a peak at 286.2 eV (Figure 3C, right), corresponding to the phenolic C─O bonds in tannic acid. Further evidence indicating covalent bonding between tannic acid and hair keratins as well as the formation of new S‐TA bonds, is given by the largely enhanced tensile strength of TA‐coated hair in comparison to untreated bleached hair. Notably, the TA‐coated hair exhibited a significant improvement in tensile strength, achieving a maximum value of 204 MPa, namely a 51% increase compared to the untreated hair sample (135 MPa).

Further evidence of TA binding to hair's keratin had been reported by Cui and coworkers in China describing in 2020 hair dyeing process for naturally gray hair via the formation of metal–phenolic networks (MPNs) composed of TA or gallic acid and different metal ions.^[^ ^10^ ^]^ Compared to tannic acid (TA) (≈2.2 nm), gallic acid (GA) smaller size (≈0.75 nm) allows better penetration into hair fibers, allowing for instance robust hair dyeing with GA–Fe(II) due to the strong interactions between GA or Fe(II) and hair surface proteins driven by hydrophobic interactions and coordination bonding and the diffusion of free molecules or small complexes into the inner hair fibers, thus contributing to hair dyeing.

**Figure 4:**
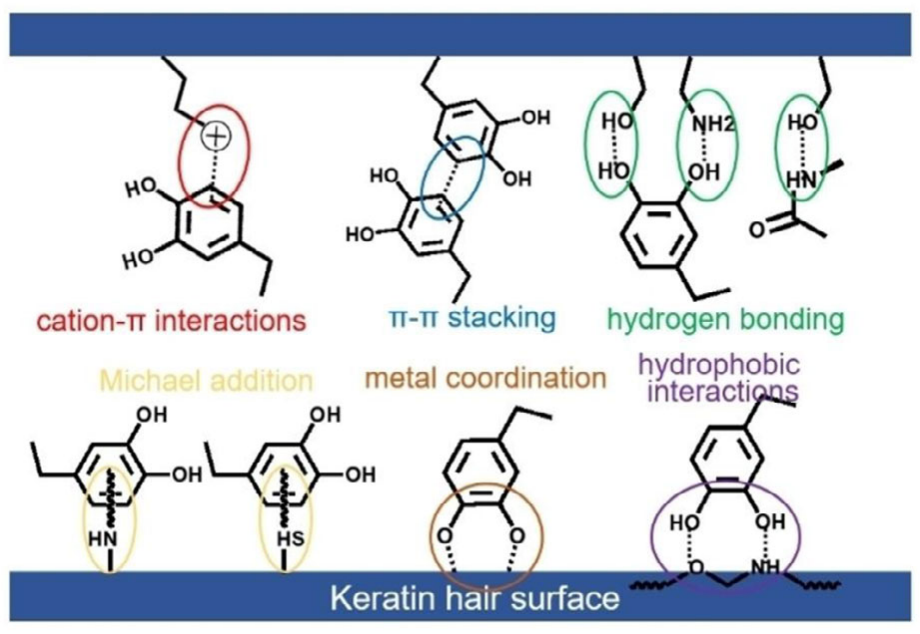
Schematic representation of possible mechanisms of MPN pigment deposition onto the hair surface. [Reproduced with kind permission from ref. [28], Copyright John Wiley & Sons, 2020].

Two years later, Wang, Qi, and coworkers in China confirmed that metal‐doped tannic acid and gallic acid MPN pigments coat and bind the hair fibers through metal coordination, cation–π interactions, and Michael addition (**Figure** **4**), with GA+TA−Fe (II) pigment showing the best hair dyeing stability.^[^ ^28^ ^]^

The SEM photographs of hair dyed with GA+TA−Fe (II) pigment before and after 20 consecutive washing cycles showed no changes in uniform nanoparticle size of ≈20 nm and morphology. The small difference in the UV spectrum before and after washing also confirmed that the substance did not change. Similarly, no changes were observed in the mass spectrometry molecular weight and UV spectrum of the product before and after washing).

The excellent stability of the product leads to the excellent effect of hair dyeing. The hair dyed by doping GA+TA had the best stability and color fastness, with a modest dyeing decrease after prolonged washing showing evidence of the new dyeing method potential for practical application. Hair dyeing with these MPNs is carried out at 25 °C under weak alkaline conditions (pH 10), without any aromatic amine such as widely employed *p*‐phenylenediamine, ammonia, oxidants, or color developers. Furthermore, the team reported the excellent biocompatibility of GA+TA−Fe(II) pigment reporting the lack of cytotoxicity of the pigment for embryonic kidney cell 293 (HEK293T) treated *in vitro* with the GA+TA−Fe(II) pigment at increasing concentrations (1–15 nM up to 18 h incubation). As mentioned in the introduction, in 2020 industrial and academic chemists based in Germany had reported that hair tresses pre‐bleached 3 times treated with 100 mL vegetable tannin solution (mimosa powder extract dissolved in water) and Fe(II)‐lactate for 30 min resulted in excellent dyeing of bleached hair.^[^ ^9^ ^]^

**Figure 5:**
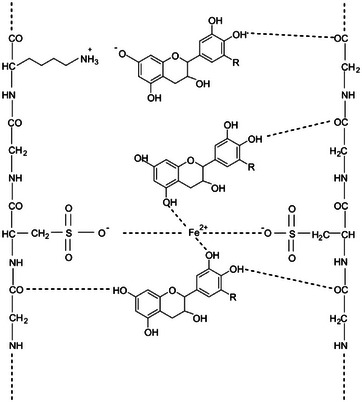
Plausible interactions of keratinous fibre with tannin–mordant complex [Reproduced with kind permission from ref. [9], Copyright John Wiley & Sons, 2020].

The bleaching process affects the keratinous structure of the hair fibre and leads to the oxidative degradation of cystine into the formation to sulphonic acid.^[^ ^29^ ^]^ The team ascribed the outcome to “the strong interactions between the sulphonic acid groups in the keratinous structure with the mordant and the hydroxyl groups of tannin molecules” (**Figure** **5**).^[^ ^9^ ^]^ The study concluded that the hair structure must be opened before vegetable dyeing to facilitate the tannin–mordant complexation mechanism.

## 4 Conclusion

In summary, both hydrolyzable and condensed tannins are rapidily emerging as key active ingredients in widely different cosmeceutical products especially suitable for skin and hair care. Said new uses of tannins in cosmeceutical products opened to this old bioproduct an affluent market in which tannins are used as bioactive ingredients of effective and health‐beneficial formulations. In said formulation, tannins successfully replace harmful chemicals associated to significant health and safety risks such as, *p*‐phenylenediamine, ammonia, oxidants, or color developers.

Recounting the case of tannin in hair care originally patented in 1982^[^ ^22^ ^]^ that had to wait the early 2010s to be rediscovered, expanded, and eventually find practical application,^[^ ^23^ ^]^ this study shows how the use of tannin in cosmetics is another case of rediscovery of chemistry research. As noted by Lévy‐Leblond, science does not develop in a linear fashion but rather goes through frequent revival of interest in areas of research considered closed by rediscovering scientific developments dating back decades.^[^ ^30^ ^]^ The case of tannin, for over two thousand years used in animal skin tannage, and then suddenly rediscovered to find completely new applications in animal feeding and as biostimulant and biopesticide in agriculture in the late 1990s and the early 2000s,^[^ ^3^ ^]^ is an eminent example of innovation in chemistry that may be used to renew researcher training, replacing the obsolete approach based on studying contemporary science alone.^[^ ^30^ ^]^

Said rediscovery of tannin as a natural product having many more potential (and actual) applications than traditional skin tannage and as a clarifying agent in winemaking, led to the introduction of the first new tannin isolation methods based on membrane technology;^[^ ^31^ ^]^ and subsequently to extraction based on hydrodynamic cavitation at room temperature that led to dramatically lower extraction costs and enhanced product quality due to substantial lower extraction temperature and extraction time.^[^ ^32^ ^]^ This is relevant to new cosmeceutical and nutraceutical uses of tannin because all these advanced applications require the employment of standardized extracts of known composition, rich in active tannins, and preferably free of extraction by‐products. Indeed, the aforementioned company that introduced the “Taninoplastia” in 2014 launched a product with a substantially higher tannin concentration (“Blue Gold Premium”). Indirectly, thus, this study shows how progress in green extraction and purification technology has been instrumental to achieve the first cosmeceutical applications of tannins.

Said advances hold large potential toward global health. As mentioned in the introduction, both hydrolyzable ^[^ ^5^ ^]^ and condensed^[^ ^6^ ^]^ tannins exhibit astringent, antimicrobial, antioxidant, anti‐inflammatory, and anti‐carcinogenic bioactivity. The replacement of toxic ingredients in hair dyeing formulations as well as of harmful ingredients in hair straightener—formulations is of global health relevance. Worldwide, indeed, according to a survey conducted in 2017 ≈40 percent of women worldwide claimed to use hair straightener (a five percent increase from 2015).^[^ ^33^ ^]^ Since then, the percentage has further increased. The global hair styling tools market size was $30.09 billion in 2019 and is projected to reach $51.44 billion by 2032.^[^ ^34^ ^]^

There are a number of hair straightening and hair dyeing formulations commercially available today, but better and safer formulations are urgently needed. Both hair dyeing using conventional aromatic amines and hair relaxer creams present health and safety risks. In the case of hair dyeing in specific subpopulations, a positive association between hair dye use and cancer occurrence has been reported.^[^ ^35^ ^]^

A study on the adverse effects of hair straightening products carried out in India in 2013 on 90 subjects, found that ≈96% of them had adverse effects, leading scholars to conclude that “it is necessary to make available a less harmful chemical hair relaxer to the society.”^[^ ^36^ ^]^ In 2022, researchers at the US National Institutes of Health (NIH) published a study that found women who used hair relaxers more than four times a year were at a higher risk of uterine cancer.^[^ ^37^ ^]^ Since then, numerous natural product‐based hair dyers have been commercialized.^[^ ^12^ ^]^ The global natural hair dye market size was worth $10.3 billion in 2023 and is expected to reach $23.3 billion by 2033, growing at a compound annual growth rate 8.5% from 2024 to 2033.^[^ ^38^ ^]^

Tannin‐based hair dyes and hair‐relaxing formulations are ideally suited to replace synthetic molecules employed in conventional formulations. Indeed, the tannin‐based “Taninoplastia” was quickly adopted by many hair and beauty salons worldwide. The company that patented it advertises its tannin‐based formulations as capable to “revitalize, hydrate and strengthen the health and integrity of the hair strands.”^[^ ^39^ ^]^ The three‐step procedure (hair is washed with a sulphate‐free shampoo, followed by drying, and application of the mixture containing tannins strand by strand, massaging the hair and scalp to enhance penetration of tannin in the–keratin fibers) lasts ≈180 min.^[^ ^39^ ^]^ Perhaps not surprisingly, given the knowledge accumulated on tannin as hair protecting and relaxing agent, the same company offers “Tanino colors,” namely a family of hair dyes in which the “tannin's main function is to push actives in the formula to the hair, which improves absorption and adherence”^[^ ^40^ ^]^ with the use of tannin “dramatically decreasing the proportion of ammonia in the formula, while its agents restructure and agglutinate the fibers promoting cortex reparation.”^[^ ^40^ ^]^

The market is already developed and includes already several manufacturers of tannin‐based hair relaxing formulations. For example, a commercial formulation 1 L in volume combining grape‐derived tannins and oils sold in Europe is advertised as capable “to fix keratin and protein in hair strands, resulting in a smoother, shinier and more manageable appearance” requiring an application time lasting just 10 to 20 min.^[^ ^41^ ^]^

An online search on the World Wide Web with the query “Taninoplastia” carried out in early 2025 on a comprehensive search engine returned 383,000 results.^[^ ^42^ ^]^ However, a search with the same query on Google Scholar, a comprehensive research database, returned only 8 results,^[^ ^43^ ^]^ all of them in Spanish, consisting of links to university repositories containing the Master or doctoral theses in which students attempted to study the effects of tannins on hair care.

This study fills a gap in the literature. Its publication will hopefully accelerate the uptake of tannins as cosmeceutical ingredients in cosmetic products massively employed such as hair dyes and hair relaxers, eventually replacing harmful chemicals with a class of natural products imparted with broad‐scope health‐beneficial properties.^[^ ^5^, ^6^ ^]^

## Conflict of Interest

The authors declare no conflict of interest.
